# Insect defoliation modulates influence of climate on the growth of tree species in the boreal mixed forests of eastern Canada

**DOI:** 10.1002/ece3.8656

**Published:** 2022-03-18

**Authors:** Emmanuel Amoah Boakye, Daniel Houle, Yves Bergeron, Martin P. Girardin, Igor Drobyshev

**Affiliations:** ^1^ Chaire industrielle CRSNG‐UQAT‐UQAM en aménagement forestier durable Institut de Recherche Sur Les Forêts Université du Québec en Abitibi‐Témiscamingue (UQAT) Rouyn‐Noranda Québec Canada; ^2^ Ministère des Forêts, de la Faune et des Parcs Direction de la recherché forestière Québec Québec Canada; ^3^ Ouranos Climate Change Consortium Montréal Québec Canada; ^4^ Forest Research Centre Université du Québec à Montréal Montréal Québec Canada; ^5^ Natural Resources Canada Canadian Forest Service Laurentian Forestry Centre Québec Québec Canada; ^6^ Southern Swedish Forest Research Centre Swedish University of Agricultural Sciences Alnarp Sweden

**Keywords:** boreal mixedwoods, climate change, dendrochronology, hardwoods, insect outbreak, natural disturbances

## Abstract

Increasing air temperatures and changing precipitation patterns due to climate change can affect tree growth in boreal forests. Periodic insect outbreaks affect the growth trajectory of trees, making it difficult to quantify the climate signal in growth dynamics at scales longer than a year. We studied climate‐driven growth trends and the influence of spruce budworm (*Choristoneura fumiferana* Clem.) outbreaks on these trends by analyzing the basal area increment (BAI) of 2058 trees of *Abies balsamea* (L.) Mill., *Picea glauca* (Moench) Voss, *Thuja occidentalis* L., *Populus tremuloides* Michx., and *Betula papyrifera* Marsh, which co‐occurs in the boreal mixedwood forests of western Quebec. We used a generalized additive mixed model (GAMM) to analyze species‐specific trends in BAI dynamics from 1967 to 1991. The model relied on tree size, cambial age, degree of spruce budworm defoliation, and seasonal climatic variables. Overall, we observed a decreasing growth rate of the spruce budworm host species, *A*. *balsamea* and *P*. *glauca* between 1967 and 1991, and an increasing growth rate for the non‐host, *P*. *tremuloides*, *B*. *papyrifera*, and *T*. *occidentalis*. Our results suggest that insect outbreaks may offset growth increases resulting from a warmer climate. The observation warrants the inclusion of the spruce budworm defoliation into models predicting future forest productivity.

## Graphical abstract

Positive effects of a warmer climate on tree growth may be offset by insect outbreaks. Non‐host trees are most likely to benefit from climate warming. Impact of the insect outbreaks extends over most of the trees’ lifespan.

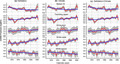

## 1 INTRODUCTION

Forests in the northern latitudes store nearly half of the global forest carbon and play a central role in the global carbon cycle (Pan et al., 2011). Changes in tree growth rates can affect the net carbon uptake and feedback to the planetary biogeochemical cycles, by altering the concentration of the atmospheric carbon dioxide (Bastin et al., 2019; Silva et al., 2010). Increases in average mean temperatures, changes in rainfall patterns, and extreme weather events since the early twentieth century have already been documented to impact the growth rates of many tree species across the boreal zone (Babst et al., 2019; Girardin et al., 2016; Lloyd & Bunn, 2007). These impacts result from the direct forcing of climate changes upon the tree physiology (e.g., the timing of budset, photosynthesis, and respiration rates) and the indirect interactions with natural disturbances, such as insect and pathogen outbreaks, fires, and windstorms (Pureswaran et al., 2015). It is anticipated that continuing climate warming will affect the natural disturbance regimes in the boreal biome, indirectly impacting tree growth (Boucher et al., 2018).

In the eastern Canadian boreal mixedwoods, coniferous and hardwood species may differ in their capacity to respond to climate change. Hardwood trees may be more adaptable to a future warmer climate, as compared to coniferous trees, due to their larger vessel diameter and deeper rooting depth allowing for more efficient water uptake (Cahoon et al., 2018; Carnicer et al., 2013). Faster growth of hardwoods under warmer climates has been suggested (Cahoon et al., 2018; Di Filippo et al., 2015; Way & Oren, 2010). The hardwoods, however, maintain a higher rate of stomatal conductance and, therefore, are more vulnerable to drought‐induced xylem cavitation and embolism than the conifers with their narrower tracheids (Brodribb et al., 2014; Cahoon et al., 2018; McDowell et al., 2008). Anatomical differences among these species may, therefore, lead to a variation in trees’ response to climate.

Spruce budworm (*Choristoneura fumiferana* Clem.) is one of the major defoliating insects of balsam fir (*Abies balsamea* [L.] Mill.) and white spruce (*Picea glauca* [Moench] Voss) in eastern Canada. The insect usually kills the host trees and those that survive the outbreak experience significant depletion of carbohydrate reserves, leading to growth reduction (Cooke & Lorenzetti, 2006; Morin, 1994; Morin et al., 1993). In the mixedwood stands, however, the diversity of the forest canopy reduces the damage by species‐specific insects due to spatial resource (Jactel et al., 2021; Poeydebat et al., 2021). In the less diverse forests, the death or the growth decline of the host trees may enhance the availability of resources, such as sunlight, nutrients, and water to non‐hosts, such as trembling aspen (*Populus tremuloides* Michx.) and white birch (*Betula papyrifera* Marsh.). It follows that biotic agents can attenuate or exacerbate climate change effects on growth in both host and non‐host trees (Bergeron et al., 2002).

There are indications that the current rate of climate warming exceeds the natural adaptability of forests to environmental changes, which may impact tree growth and lead to changes in insect activity, shifts in species ranges, and modify the composition of forest communities (Astrup et al., 2018; Gauthier et al., 2015). Current management strategies may not be able to effectively deal with the forest changes related to the climate warming, warrantying adjustment of timber harvesting and production strategies. These adjustments could include minimizing disturbance during harvesting activities and reducing the susceptibility of the forest stands to water stress and wildfire (Gauthier et al., 2015; Government of Québec, 2015; Keenan, 2015).

To quantify the effects of climate and insect outbreaks on growth, we studied the growth response of dominant species in boreal mixedwood forests in western Quebec. Since 1950, this region has been experiencing both rising temperatures and increasing precipitation (Girardin et al., 2014; Girardin & Wotton, 2009; Price et al., 2013). During the second half of the twentieth century, extensive outbreaks of spruce budworm have been documented in the conifer‐dominated forests of the region, especially during 1972–1987 period (Morin et al., 1993). The spruce budworm outbreaks have been reported to change the structure and composition of the boreal forests (Labadie et al., 2021; Navarro et al., 2018). The region is, therefore, a good location to study the effect of climate‐outbreak interaction upon tree growth.

To study tree growth patterns affected by climate and insect outbreaks, we used 2058 tree‐ring chronologies of *Abies balsamea* (L.) Mill., *Picea glauca* (Moench) Voss, *Thuja occidentalis* L., *Populus tremuloides* Michx, and *Betula papyrifera* Marsh growing on the interface between temperate and boreal mixedwoods in Western Quebec. Specifically, we hypothesized that (H1) the increase of temperatures and changing precipitation patterns generally favored tree growth, and (H2) the combined impacts of temperature and precipitation changes, as well as the emergence of insect epidemics, were overall more positive for the growth of non‐host trees and more negative for hosts.

## 2 MATERIALS AND METHODS

### 2.1 Study area

**FIGURE 1:**
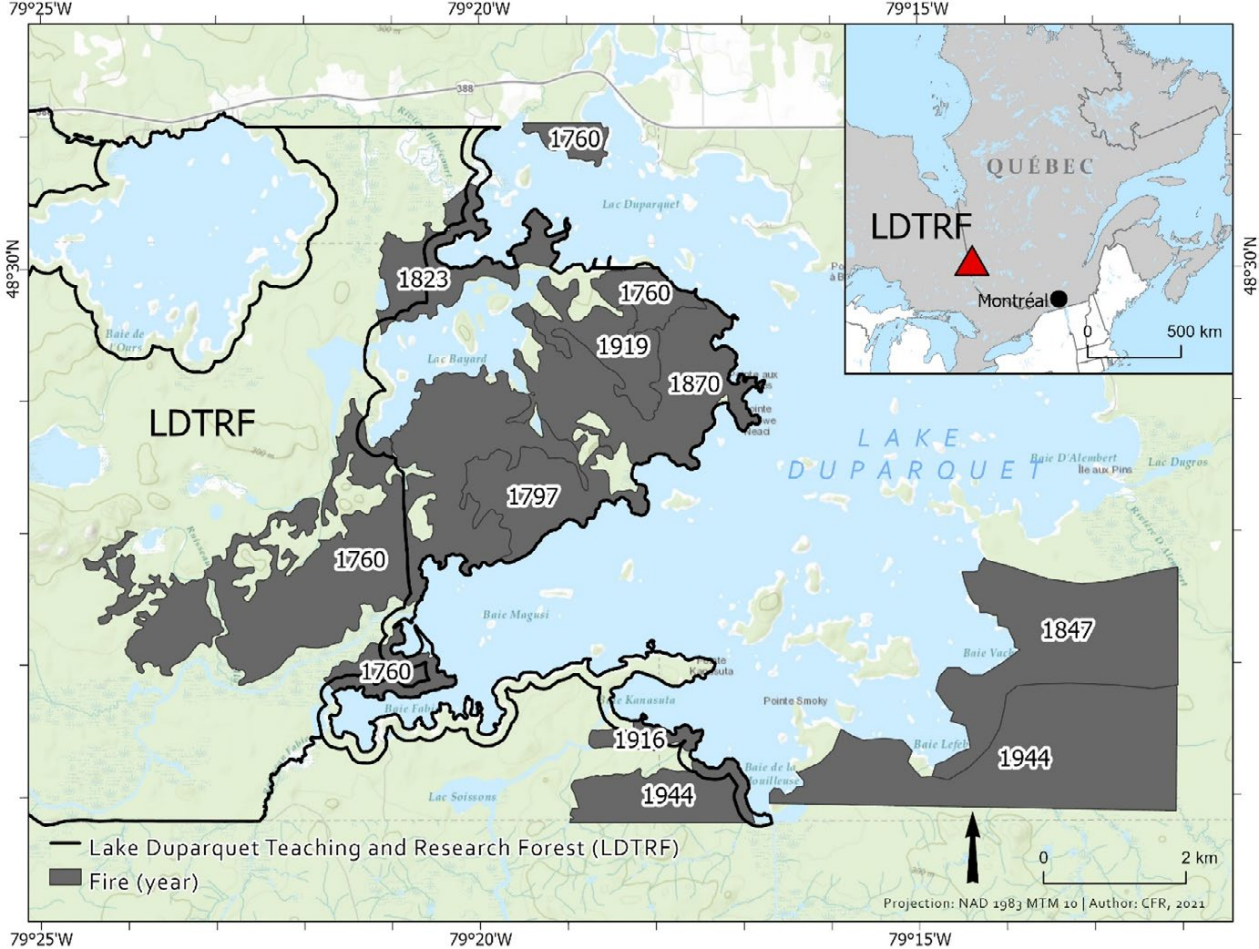
Map of the study area, Lake Duparquet Teaching and Research Forest (LDTRF) in Western Quebec, Canada

We conducted the study in mixedwood stands growing in the balsam fir‐white birch domain in the Abitibi region of Quebec, Eastern Canada (Figure 1). The study was located in the Research and Teaching Forest of Lake Duparquet (48.46 N, 79.24 W) at 270–275 m above sea level. The long‐term average annual temperature between 1961 and 1990 was 0.8°C. Also, the annual precipitation was, on average, 856.8 mm (Bergeron, 2000). The main soil type is clay (Aussenac et al., 2017a; Bergeron, 2000). The dominant canopy species include white birch (*Betula papyrifera Marsh*.), trembling aspen (*Populus tremuloides* Michx.), balsam fir (*Abies balsamea* [L.] Mill.), white spruce (*Picea glauca* [Moench] Voss), and eastern white cedar (*Thuja occidentalis* L.) (Bergeron et al., 2014). The stands originated from seven different fires that occurred in 1760, 1797, 1823, 1847, 1870, 1916, and 1944 (Leduc et al., 2020). The studied stands have not been affected by logging and can be viewed as a result of natural succession (Bergeron et al., 2002).

### 2.2 Data collection and preparation

We studied balsam fir, white spruce, eastern white cedar, trembling aspen, and white birch. These species were selected because they are the dominant species in the forest successional dynamics of Lake Duparquet. Younger forest stands (74 and 120 years) were dominated by trembling aspen and white birch, mid‐aged stands (143 and 167 years) were dominated by balsam fir and white spruce, whereas older stands (193 and 230 years) were dominated by white cedar. Trembling aspen (10 m^2^ ha^−1^) had the highest basal area in the forest stand, followed by balsam fir (9 m^2^ ha^−1^), white birch (5 m^2^ ha^−1^), white spruce (4 m^2^ ha^−1^), and the least being white cedar (3 m^2^ ha^−1^) (Bergeron, 2000).

We retrieved dated chronologies of the species from the tree‐ring databank of the dendrochronological laboratory of the Université du Québec en Abitibi‐Témiscamingue. The tree‐ring data were derived from sampling the cross‐section of trees at the height of 1m with a diameter ≥2 cm in 1990 (Bergeron, 2000) and increment cores taken at the height of 1.3 m in 2012 and 2013 (Aussenac et al., 2017b). The samples were dried, glued to a wooden holder, and sanded with sandpaper to obtain a smooth surface. Ring boundaries were first detected and identified under ×40 magnification, cross‐dated through the use of pointer years, and then measured to the nearest 0.01 mm precision with the WinDendro Image Analysis System (Regent Instruments Inc.) and Henson incremental measuring device (Mission Viejo, California, USA). The total number of tree‐ring series for all species was 2058 (Table 1, Figure S1).

**TABLE 1:** Characteristics of sampled trees of the studied species in Lake Duparquet Teaching and Research Forest of Western Quebec

Species characteristics	White cedar	White spruce	Balsam fir	White birch	Trembling aspen
Leaf type	Conifer	Conifer	Conifer	Deciduous	Deciduous
Total sample of trees	481	136	856	173	412
Inter‐series correlation	0.81	0.75	0.73	0.87	0.71
Expressed population signal	0.91	0.96	0.95	0.92	0.88
Max tree age	183	162	151	224	87
Basal area increment ± SD, cm^2^ year^−1^	3.64 ± 4.7	6.61 ± 5.60	2.26 ± 3.60	7.61 ± 9.6	5.24 ± 7.65
Variance measure	0.39	0.14	0.38	0.44	0.36

Ring width does not give a correct approximation of growth trends, due to its inability to account for the changes in trunk diameter throughout the tree lifespan (Biondi & Qeadan, 2008; Sullivan et al., 2016). Accordingly, we converted ring width into basal area increment (BAI) to improve the representation of the growth rate and biomass accumulation with the formula for the area of a circle: $\text{BAI =}\;\pi \left(R_{t}^{2}‐R_{t‐1}^{2}\right)$, where *R* is tree radius, *t* is the year when the ring was formed, and *π* is 3.142. The function *bai*.*out* in the R package *dplR* was used to compute BAI (Bunn, 2008; R Core Team, 2018). We expressed annual growth rates as cm^2^ year^−1^. Inter‐series correlation and expressed population signal (EPS) were used to assess the strength of the population‐wide growth signal in the chronologies (Table 1). EPS values for all species were above 0.85, indicating that the population signal in chronologies was sufficiently strong (Wigley et al., 1984). Rings that formed during the first 10 years of growth were removed from analyses, since at that age the climatic signal in growth tends to be weak, due to the strong and varying effects on growth, exercised by the local conditions, such as snow load on the crown, browsing, light/drought conditions, and proximity to taller trees (Marchand et al., 2019). Figure S2 shows the temporal pattern of the BAI for each species between 1800 and 2013.

We compiled historical records of defoliation severity that were incurred by spruce budworm for the study area between 1967 and 1991 from Quebec's annual provincial surveys of the Ministère des Forêts, de la Faune et des Parcs (MFFP). These annual surveys provide aerial estimates of the intensities of damage caused by spruce budworms across Québec. The intensity of the damage is expressed in percentage with 0 indicating no defoliation and 100 indicating severe defoliation (MFFP, 2018). Since the trembling aspen and white birch chronologies extended only to 1991, we limited the analyses for all studied species to 1967–1991.

**FIGURE 2:**
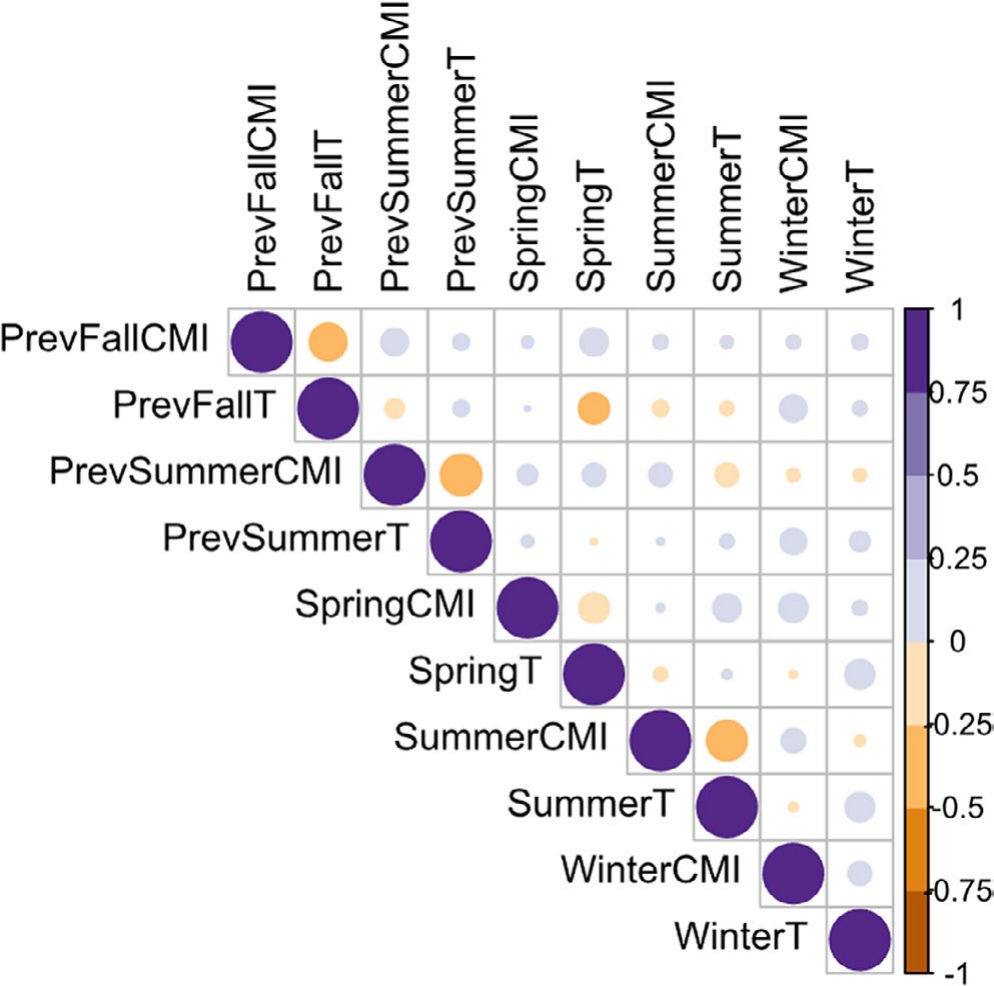
Pearson correlation matrix between seasonal climate variables. Suffixes CMI and T denote climate moisture index and temperature, respectively. The prefix Prev denotes previous

We obtained a historical temperature and climate moisture index (CMI) for the study site. CMI characterizes the available moisture and reflects the balance of monthly potential evapotranspiration (PET) and monthly precipitation (Preci) in mm of water, CMI = Preci*_i_*–PET*_i_*, over a time period *i* (Berner et al., 2017; Hogg, 1997). The CMI is typically well correlated with tree growth in boreal and temperate forest ecosystems (Berner et al., 2017). Potential evapotranspiration was estimated with the R package *SPEI*. The package used the Penman–Monteith algorithm with inputs of daily minimum and maximum temperatures averaged per month, latitude, monthly incoming solar radiation, monthly temperature at dew point, and altitude (Vicente‐Serrano et al., 2010). The temperature and CMI were calculated using the *BioSIM 10* software (Régnière et al., 2014), operating on the Environment Canada's weather station network. We obtained temperature and CMI chronologies for three seasons of the focal year: winter (December to February), spring (March to May), and summer (June–August). We also obtained previous summer and fall CMI and temperature data to assess the influence of the previous year's weather on tree growth. In Figure 2, we show a correlation matrix of the climatic variables to evaluate the potential impact of multicollinearity on model results. In order to understand the temporal trends in climate variables, we regressed seasonal temperatures and CMI against time, using linear regression.

### 2.3 GAMM model

We used GAMM to model the basal growth rate (BAI) of tree *j* at specific year *t* as a function of the tree basal area (variable BA) and its cambial age (variable Age). To understand the influence of climate variability and insect outbreak, we included seasonal climatic variables (temperature, *T* and climate moisture index, CMI) and the spruce budworm defoliation levels (DFI) into the model. A single GAMM was fitted for all species and, therefore, species (*S*) was introduced as a factor. The global model took the following form:

(1) $$\text{log}\left({\text{BAI}}_{\mathrm{jt}}\right)=\beta \left(\text{log}\left({\text{BA}}_{\mathrm{jt}},S\right)\right)+f\left({\text{Age}}_{\mathrm{jt}},S\right)+f\left(T_{t},S\right)+f\left({\text{CMI}}_{t},S\right)+f\left({\text{DFI}}_{t},S\right)+Z_{j}+\varepsilon_{j}+{\text{AR1}}_{j}$$

where BA*_jt_* is the inner‐bark basal area of tree *j* at specific year *t* (computed as the sum of BAI of previous years), Age is the cambial age of tree *j* at year *t*, *T_t_* is the temperature at year *t*, and CMI*_t_* is the CMI at year *t*. The tree code (Z*_j_*) was used as a random effect in the GAMM. We included an auto‐regressive term (AR(1)*j*) to account for the temporal correlation between successive values of BAI data. GAMM was realized in R package *mgcv* (Wood, 2017). The inclusion of BA and Age in the model helped us increase the model realism and address low‐frequency variation in the initial data, thereby improving the model prediction skill (Dietrich & Anand, 2019).

As an initial step in the analysis of the model output, we evaluated the concurvity of the smoothing variables in the GAMM model (Figure S3). Concurvity refers to the degree to which a smooth model term that detects trends can be approximated by one or more smooth model terms (Johnston et al., 2018). It describes the instability of the estimated coefficients of the smoothing terms in GAMM models. The concurvity index was calculated on a scale of 0 to 1, with 0 indicating no concurvity and 1 indicating high concurvity (Morlini, 2006). We further tested how each climatic variable, *T_t_* and CMI*_t_*, performed separately in competing GAMMs for each species. We also tested the full GAMM model with or without random effects. The model with the lowest value of Akaike's information criterion was selected, using the R package *AICcmodavg* (Mazerolle, 2020).

The model was fit on a randomly sampled subset of 1646 trees, corresponding to 80% of all trees. The predictive capacity of the model was then validated using the remaining 20% of the data (412 trees). For validation, we computed the adjusted R^2^ and the root mean square error (RMSE) of predicted versus observed growth rates. The normality and homoscedasticity of GAMM residuals were assessed visually (Figure S4).

To estimate the effect of the insect defoliation on the growth trajectory, we predicted spruce budworm defoliation in the full GAMM model (Equation 1) by allowing DFI_t_, and *Z_j_* to vary and keeping the seasonal climatological means (*T_t_* and CMI*_t_*), BA*_jt_*, and Age*_jt_*, constant at zero. We estimated the effect of climate, by allowing variation of seasonal climate (*T_t_* and CMI*_t_*) and *Z_j_* variables and keeping DFI*_t_*, BA*_jt_*, and Age*_jt_* variables constant at zero. Finally, we predicted the growth, allowing all variables in the full version of the GAMM model to vary, by keeping their effects in the model.

To test for temporal trends in BAI chronologies of each species, we relied on a linear mixed effect models (LME), which used the BAIs predicted by the GAMM as dependent variables. In the LME, we included the calendar year (Year) of tree *j* as the fixed effect, tree identity (*Z_j_*) as the random effect, and the auto‐regressive term (AR(1)). The model took the following structure:

(2) $$\text{log}\left({\text{GAMMPredictedBAI}}_{j}\right)=\beta \left({\text{Year}}_{j}\right)+Z_{j}+\varepsilon_{j}+{\text{AR1}}_{j}$$

## 3 RESULTS

### 3.1 Climate dynamics

**FIGURE 3:**
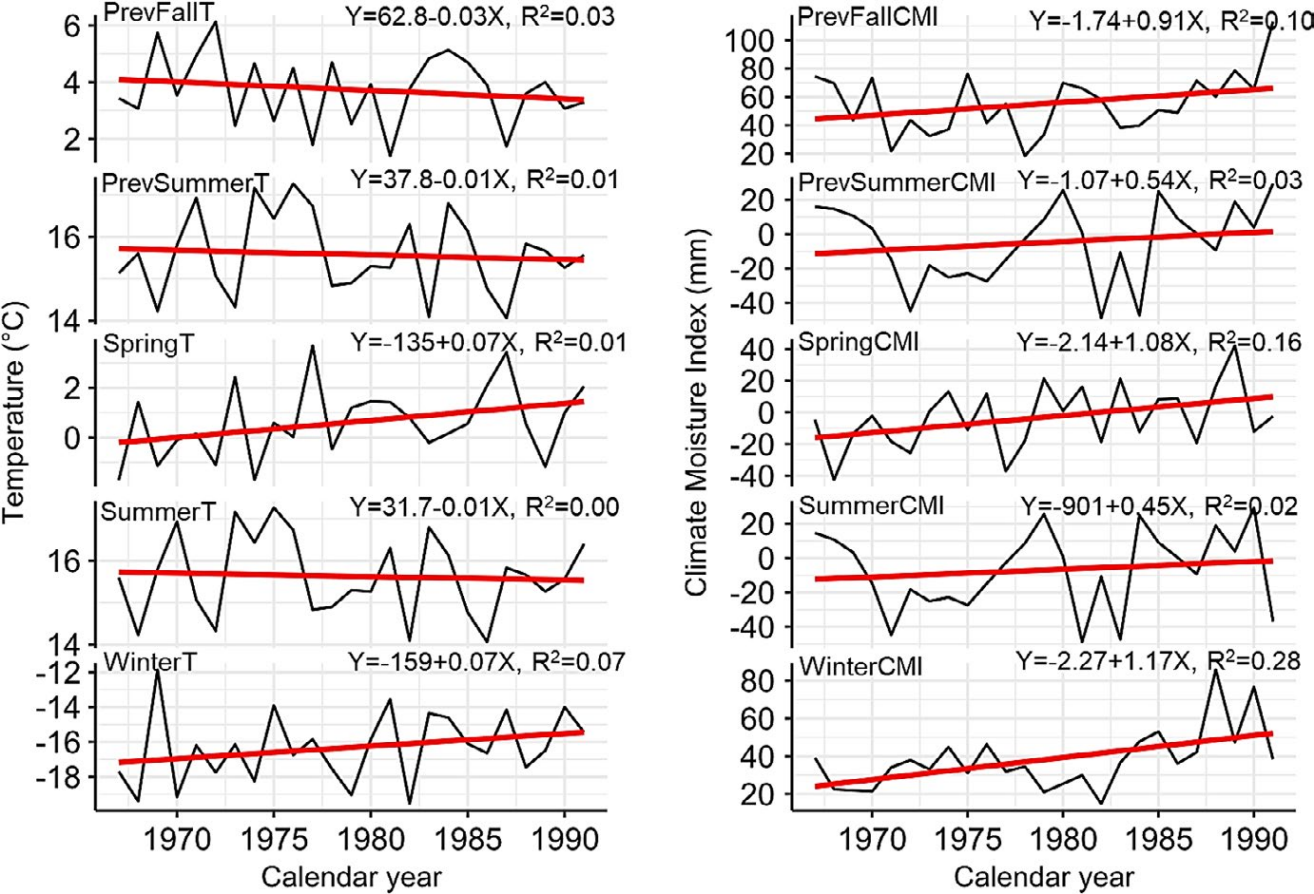
Changes in seasonal climate variables during the period between 1967 and 1991. The prefix *Prev* denotes previous, whereas the suffixes CMI and T denote climate moisture index and temperature, respectively

Regression analysis on climate variables in the study area showed that average winter and spring temperatures increased between 1967 and 1991 (Figure 3). Summer, previous fall, and previous summer temperatures visibly had little to no overall change during the period between 1967 and 1991. The seasonal CMI showed that the study area is becoming wetter, due to the increases in CMI. The correlation between the seasonal temperature and moisture index did not exceed 0.30 (Figure 2), allowing us to retain all the variables in the GAMM model.

### 3.2 GAMM growth model

The GAMM model with the lowest AIC had the following set of predictors: basal area (a proxy of tree size), cambial age, defoliation intensity, seasonal temperatures, and CMI (Table 2; Figure S4). We observed low estimated concurvity (concurvity <0.50) between the model variables, which suggests that the functional relationship between them is weak (i.e., low collinearity) (Figure S3). Overall, the proportion of variance explained by the GAMM model was 85% (Table 2). The GAMM model also showed that all variables included in the model were significant at *p* < .05.

**TABLE 2:** Summary of generalized additive mixed model of the effect of tree size, age, and seasonal climatic variables on growth rates

Characteristics	df	AICc	RMSE	*R* ^2^
(a) GAMM growth model comparison				
Full model with random factor as in Equation 1	1519.54	6778.39	0.28	.85
Model without random factor in Equation 1	128.03	11928.59	0.33	.79
Full model with all variables as in Equation 1	1519.54	6778.39	0.26	.85
Model with CMI excluded from Equation 1	1486.84	7990.28	0.42	.84
Model with T excluded from Equation 1	1473.54	7955.45	0.42	.84

(b) Summary of the full GAMM growth model (Equation 1)			
*R* ^2^ adjusted (%)	0.85		
Parametric coefficients (± SE, *p*‐value)			
Intercept	0.37 ± 0.17	0.029	
Significance of smoothing predictors (edf, *F*, *p*‐value)			
log.BA	8.81	36169.72	<.001
Cambial age (years)	9.76	1150.33	<.001
PrevSummerT (^o^C)	8.98	20.27	<.001
PrevSummerCMI (mm)	9.87	39.39	<.001
PrevFallT (^o^C)	9.49	40.79	<.001
PrevFallCMI (mm)	9.32	15.02	<.001
WinterT (^o^C)	9.01	41.07	<.001
WinterCMI (mm)	12.08	41.70	<.001
SpringT (^o^C)	8.66	9.89	<.001
SpringCMI (mm)	9.19	15.97	<.001
SummerT (^o^C)	9.65	38.08	<.001
SummerCMI (mm)	8.91	12.35	<.001
Defoliation index	9.53	36.27	<.001
Subject trees	1390.11	4.46	<.001

Note

The prefix Prev denotes previous, whereas the suffixes CMI and *T* denote climate moisture index and temperature, respectively. BA = basal area, RMSE = root mean square error, *p* values indicate the significance of the effect of variables at lower than .05.

### 3.3 Spruce budworm defoliation versus climate effects

**FIGURE 4:**
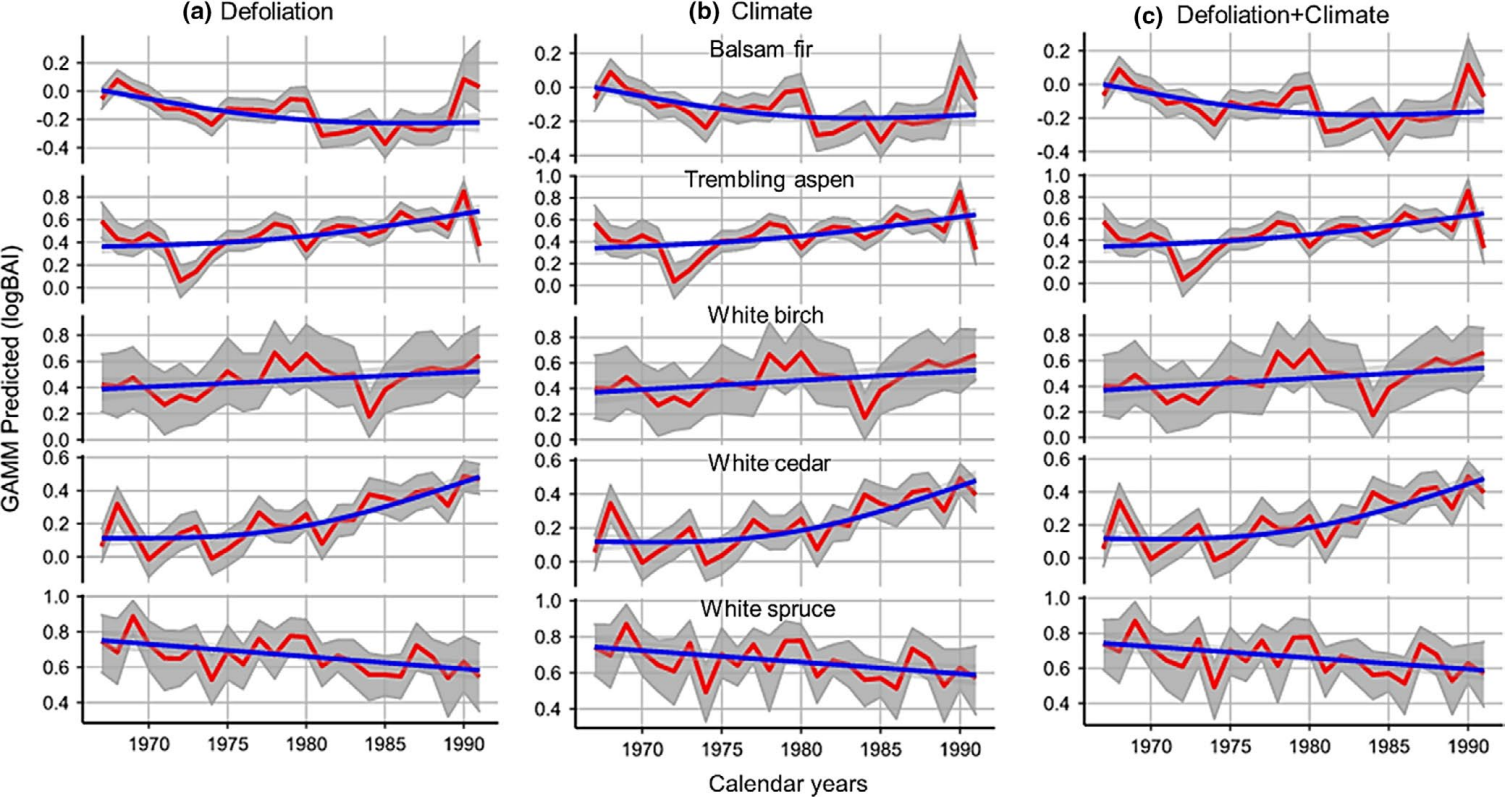
Temporal variability of predicted basal area increment (log BAI) of the species in relation to spruce budworm defoliation, climate, and the two factors combined. The Y‐axis is the log of BAI, as predicted by GAMM. Blue lines are curves representing fitted generalized additive models using a knot of 3. Red lines represent the predicted values from GAMM. Gray shading delimits the bootstrapped 95% confidence intervals. Each row of places represents a species

Temporal dynamics (Figure 4) of the tree growth showed that the non‐host of spruce budworm, trembling aspen, and white cedar grew at a slower rate between 1967 and 1980. Following 1980, the growth of the two species increased over time. White birch, on the other hand, had been increasing in growth over time between 1967 and 1991. The growth of the spruce budworm host, white spruce, decreased over time between 1967 and 1991. The growth of balsam fir, however, decreased over time between 1967 and 1985, after which growth increased slightly.

**FIGURE 5:**
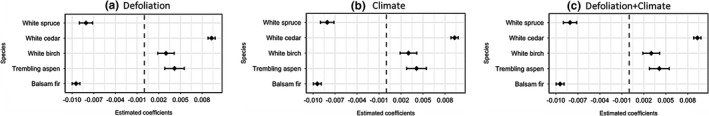
Species‐specific linear mixed model estimates of the effects of defoliation, climate, and all factors combined (full GAMM model, Equation 1) on the growth of tree species

Overall, the combined effects of the climate variability and spruce budworm defoliation negatively affected the growth of balsam fir and white spruce, and positively affected the growth of white cedar, trembling aspen, and white birch (Figure 5). Similarly, the climate variability and spruce budworm separately had a significant negative effect on the growth of white spruce and balsam fir (Figure 5). Raw BAI series revealed growth releases in non‐host species, in particular, white cedar and trembling aspen, during the suppression of the host species (white spruce and balsam fir) from the spruce budworm outbreak of 1972–1987 (Figure S2).

## 4 DISCUSSION

Our results highlight the importance of climate variability and insects in controlling tree growth in the mixedwood forests of eastern Canada. We observed a decreasing growth rate of the spruce budworm host species, balsam fir and white spruce, and an increasing growth rate of the non‐hosts, trembling aspen, white birch, and white cedar (Figures 4 and 5). Our results partially supported H1, which proposed that the increase of temperatures and changing rainfall patterns generally favored tree growth. Our results supported H2, since we observed that the combined effects of temperature and precipitation changes, as well as the emergence of insect epidemics, were overall more positive for the growth of non‐host trees and more negative for hosts. Climate warming increases moisture availability and the length of the growing season, which correlates positively with tree growth (Price et al., 2013). However, the lengthening of the growing season makes it possible for insects to produce several generations within the same season, increasing the risk of insect outbreaks and associated growth declines. The enhancement of the deciduous species growth and the reduction of conifer growth have been widely reported across the northern hemisphere, including northern Japan (Tsutom et al., 2019), eastern (Anyomi et al., 2012; Boakye et al., 2021) and western Canada (Searle & Chen, 2018; Zhang et al., 2008).

The defoliation of the spruce budworm hosts, balsam fir and white spruce, might indirectly promote the growth of non‐hosts trees, trembling aspen, white birch, and white cedar (Figures 4 and 5). Tree defoliation by the insects creates large openings, where non‐host trees likely experience reduced competition from the surviving host trees for sunlight, water, and nutrients. This promotes tree growth of the non‐host species (Anderegg et al., 2015; Bergeron, 2000; Boulanger et al., 2017). Consistent with our observations, studies have reported the spruce budworm defoliation as a factor driving an increase in the growth of non‐host species in eastern and western Canadian boreal forests (Duchesne & Ouimet, 2008; Itter et al., 2019).

The growth of the spruce budworm host species, balsam fir and white spruce (Figures 4 and 5), declined between 1967 and 1991. The pattern might be due to the higher temperatures accelerating transpiration, stomata closure, and limited access to atmospheric carbon. The growth decline of the conifers provides adequate canopy openings that promote the regeneration of the shade‐intolerant species (Anderegg et al., 2012, 2015; Barber et al., 2000). This compositional change can impact the climate system, since the reduced evergreen conifer cover will expose the landscape in winter, increasing the surface reflectance and the cooling of the boreal mixedwoods (Mcdowell et al., 2016).

The defoliation by spruce budworm might also contribute to the growth decline of the balsam fir and white spruce (Figures 4 and 5). Between 1960 and 1980, the budworm caused massive defoliation in the study area, likely leading to carbon starvation and subsequent growth decline in surviving trees (Morin et al., 1993). Mined needles have shown a reduced water transport efficiency and lower carbon absorption potential, and the affected trees have revealed a limited investment of resources into defensive compounds (Anderegg et al., 2012, 2015; Morin, 1994).

Differences in xylem anatomy, plant allometry, stomatal behavior, rooting strategies, stand density, and forest management can all modify the response of tree species to environmental variability. For example, deciduous trees such as white birch and trembling aspen have a deeper root system than conifers, enabling them to access water from deeper soil layers to better overcome seasonal water deficits, as compared to coniferous species (Mekontchou et al., 2020; Oltchev et al., 2002).

Different stand densities modulate tree response to spruce budworm outbreaks and climate variability by controlling competition between trees (De Grandpré et al., 2019; Steckel et al., 2020). Higher densities of host species and warmer climates can increase the intensity of tree defoliation, and promote growth reduction and mortality in the host trees. The resulting canopy openings will increase the availability of light and water to promote the growth of the hon‐hosts (Chavardès et al., 2021; Steckel et al., 2020).

The studied stands experienced no silvicultural treatments such as thinnings or selective fellings as the stand management has been based on the natural disturbance dynamics (Harvey, 1999).

### 4.1 Implication for forest management

Climate warming and insect outbreaks influence the growth of the studied mixedwood forests of eastern Canada. These influences have a significant and likely long‐lasting impact on the forest ecological and economic value and forest management. There is a need to develop and implement the necessary changes to mitigate the adverse effects and to take advantage of the changing environmental settings.

The decreased growth rate of the spruce budworm host species (balsam fir and white spruce) will increase the time needed for the trees to reach maturity and, subsequently, the rotation time of the stands (Bigler, 2016). An increased rotation time means that the trees could provide more long‐term storage of sequestrated carbon in the living biomass. Furthermore, if the rotation periods are increased, the economically optimal number of thinnings may increase, which could incur additional costs for the forest owners. On the other hand, extended rotation may also diversify the stand composition and promote mature and old‐forest features such as multilayered canopies, down deadwood, and standing snags which will benefit local wildlife (Martin et al., 2018).

Climate warming may increase the survival of spruce budworms, potentially leading to more frequent and/or more severe outbreaks and offsetting effects of longer rotation period on carbon storage capacity (Dymond et al., 2010; Jactel et al., 2019; Logan et al., 2003). More severe budworm outbreaks will cause higher tree mortality and greater revenue loss from forestry operations. We, however, caution against the proposed use of pesticides to control the spruce budworm due to potentially extensive and long‐term negative effects on forest ecosystems (James et al., 2017).

We expect that the growth increases among the non‐hosts of the spruce budworm, especially in trembling aspen, may contribute to a short‐term increase in the deciduousness of the mixedwood forests that may increase the production of hardwood, such as lumber and wood biomass used in the production of pulp and paper (Balatinecz & Kretschmann, 2001). However, the rapid growth of the deciduous hardwoods would mean that the trees will mature and die earlier, which may lower the residence time of carbon in their biomass (Büntgen et al., 2019). The earlier maturity of the trees may also ensure a faster transition to a forest dominated by the slow‐growing conifers (Bigler, 2016; Leduc et al., 2020). Jointly, these effects will likely affect the rotation periods for the deciduous and coniferous components of mixed stands.

## CONFLICT OF INTEREST

None.

## AUTHOR CONTRIBUTIONS

**Emmanuel Amoah Boakye:** Conceptualization (equal); Formal analysis (equal); Methodology (equal). **Daniel Houle:** Supervision (equal); Writing – review & editing (equal). **Yves Bergeron:** Supervision (equal); Writing – review & editing (equal). **Martin P. Girardin:** Supervision (equal); Writing – review & editing (equal). **Igor Drobyshev:** Supervision (equal); Writing – review & editing (equal).

## Supporting information

Appendix S1

## Data Availability

Data available from the Repositories: http://dx.doi.org/10.5061/dryad.55gb7 and https://doi.org/10.1002/ecy.3306
