# Supplementary material for: Insect defoliation modulates influence of climate on the growth of tree species in the boreal mixed forests of eastern Canada
Source: Ecol Evol. 2022 Mar 18;12(3):e8656. doi: 10.1002/ece3.8656 (PMC8932224; doi:10.1002/ece3.8656)
Supplement: Supplementary file 1 — Appendix S1 [file ECE3-12-e8656-s001.docx]

Supporting Information

Insect defoliation modulates influence of climate on the growth of tree species in the boreal mixed forests of eastern Canada

Emmanuel Amoah Boakye^1^, Daniel Houle^2,3^, Yves Bergeron^1,4^, Martin P. Girardin^5^, Igor Drobyshev^1,6^

^1^ Chaire industrielle CRSNG-UQAT-UQAM en aménagement forestier durable, Institut de Recherche Sur Les Forêts, Université du Québec en Abitibi-Témiscamingue (UQAT), 445 boul. de l′ Université, Rouyn-Noranda, Québec, J9X 5E4, Canada.

^2^ Ministère des Forêts, de la Faune et des Parcs, Direction de la recherché forestière, Québec, QC G1P 3W8, Canada

^3^Ouranos Climate Change Consortium, Montréal, QC H3A 1B9, Canada.

^4^ Forest Research Centre, Université du Québec à Montréal, Montréal, Québec, Canada.

^5^ Natural Resources Canada, Canadian Forest Service, Laurentian Forestry Centre, 1055 du P.E.P.S, P.O. Box 10380, Stn. Sainte-Foy, Québec, QC, G1V 4C7, Canada.

^6^ Southern Swedish Forest Research Centre, Swedish University of Agricultural Sciences, P.O. Box 49, 230 53, Alnarp Sweden.

Corresponding author is Igor Drobyshev, [igor.drobyshev@uqat.ca](mailto:igor.drobyshev@uqat.ca)

| White spruce | White cedar |
| --- | --- |
| 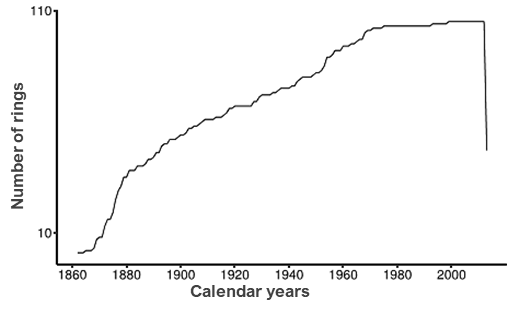 | 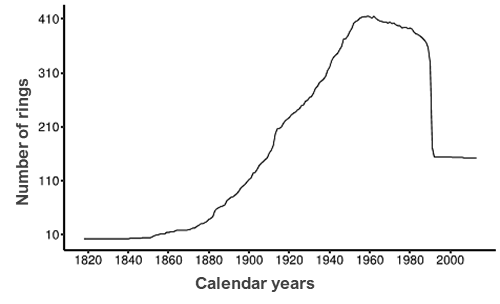 |
| White birch | Trembling aspen |
| 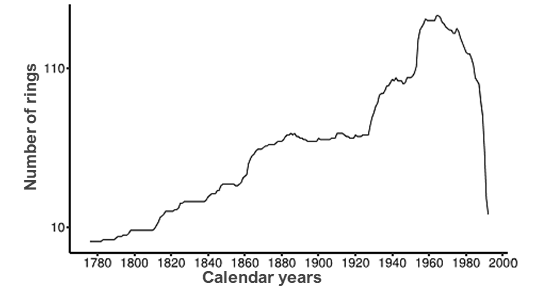 | 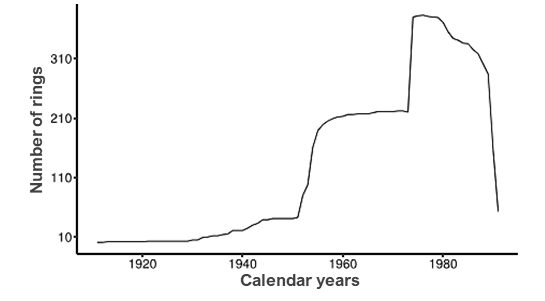 |
| Balsam fir |  |
| 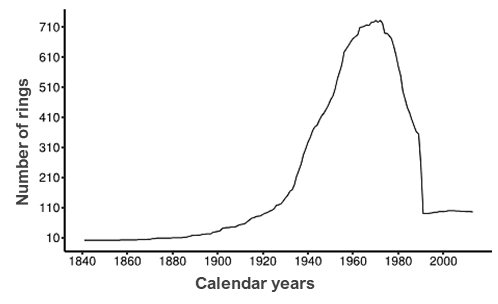 |  |
|  |  |

Figure S1. Sample depth, i.e. the number of tree rings analyzed as a function of time and species


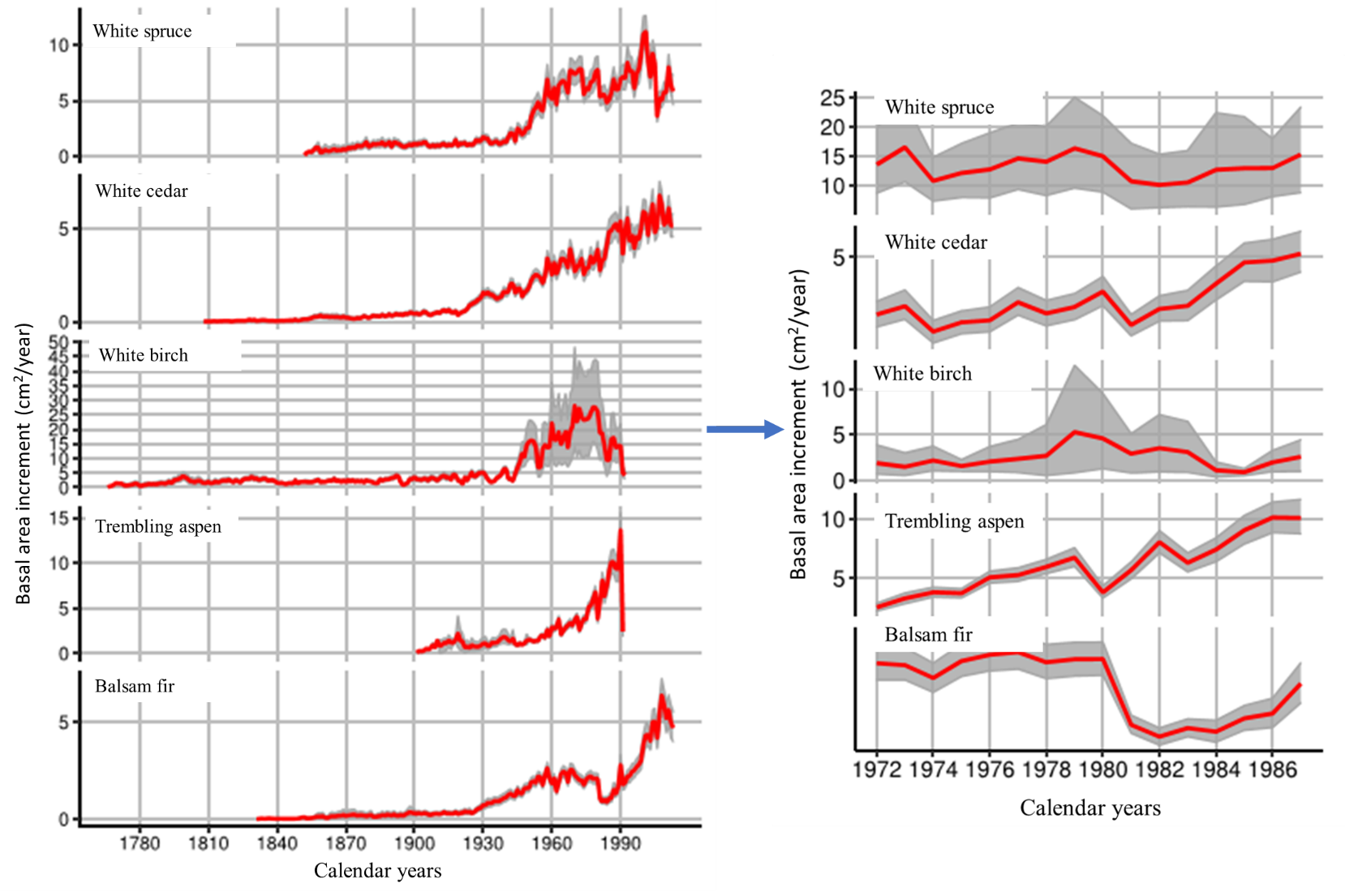


Figure S2. Temporal pattern of basal area increment of each tree species over their lifespan. The graph on the left shows the growth pattern of the entire chronology, and the graph on the right shows the period between 1972-1987, when spruce budworm defoliation was intensive.


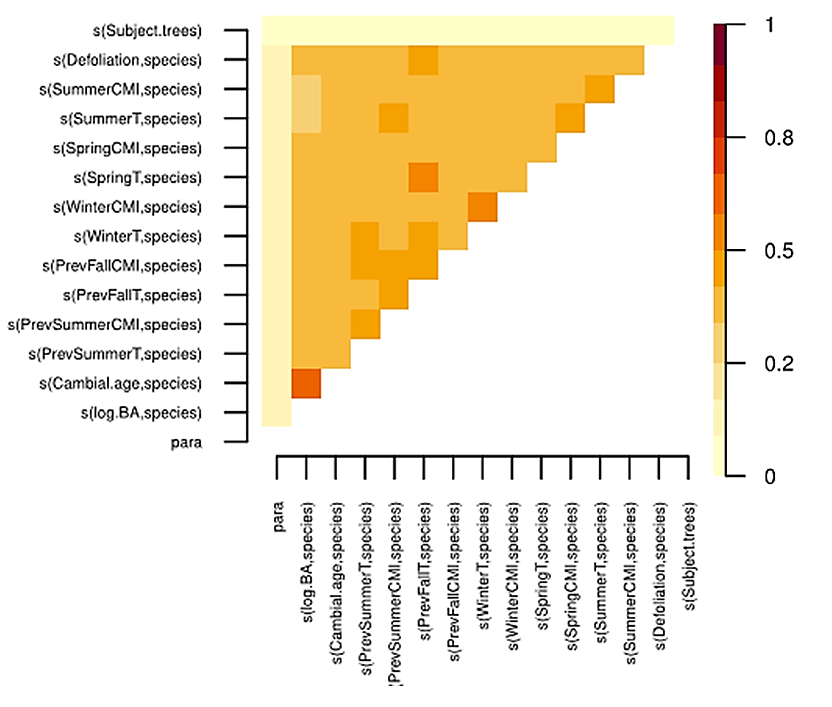


Figure S3. Estimated pairwise concurvity values between smoother terms in the model predicting trees growth rates.

Pairwise concurvity with 0 and 1 indicates low and high concurvities respectively. CMI and T denote Climate Moisture Index and Temperature respectively


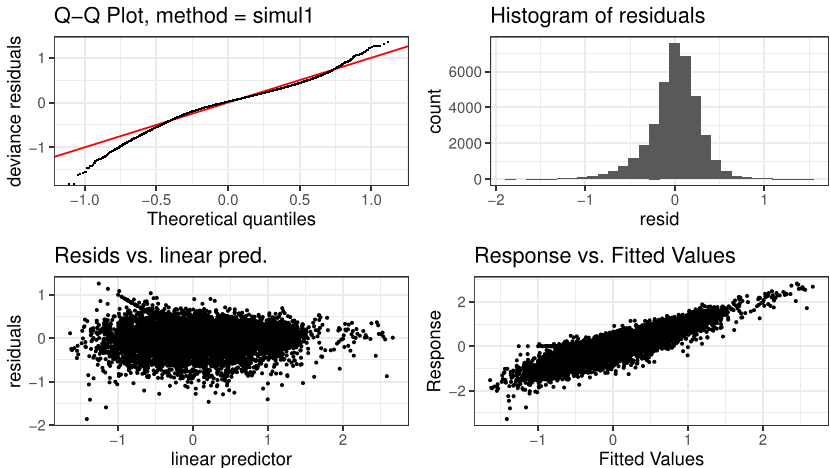


Figure S4. GAMM checking plot for the full model fit for the basal area growth rates
